# Professionalism and inter-communication skills (ICS): a multi-site validity study assessing proficiency in core competencies and milestones in medical learners

**DOI:** 10.1186/s12909-020-02290-3

**Published:** 2020-10-14

**Authors:** Abd Moain Abu Dabrh, Thomas A. Waller, Robert P. Bonacci, Anem J. Nawaz, Joshua J. Keith, Anjali Agarwal, John Merfeld, Terri Nordin, Mary Michelle Winscott, Thomas E. Belda, Mohammad Hassan Murad, Sally Ann L. Pantin, Lawrence W. Steinkraus, Thomas J. Grau, Kurt B. Angstman

**Affiliations:** 1 Department of Family Medicine, Mayo Clinic Florida, Jacksonville, FL USA; 2 Integrative Medicine and Health, Department of General Internal Medicine, Mayo clinic, Jacksonville, FL USA; 3 Department of Family Medicine, Mayo Clinic, Rochester, MN USA; 4 Department of Family Medicine, Mayo Clinic Health System, La Crosse, WI USA; 5 Department of Family Medicine, Mayo Clinic health System, Eau Claire, WI USA; 6 Department of Family Medicine, Mayo Clinic, Phoenix, AZ USA; 7 Center for Simulation, Mayo Clinic, Rochester, MN USA; 8 Division of Preventive Medicine, Mayo Clinic, Rochester, MN USA

**Keywords:** Communication skills, Professionalism, Core competencies, Milestones, Assessment, Validity, InCoPrA, Medical learners, Simulation

## Abstract

**Background:**

Interpersonal and Communication Skills (ICS) and Professionalism milestones are challenging to evaluate during medical training. Paucity in proficiency, direction and validity evidence of assessment tools of these milestones warrants further research. We validated the reliability of the previously-piloted *Instrument for Communication skills and Professionalism Assessment* (InCoPrA**)** in medical learners.

**Methods:**

This validity approach was guided by the rigorous Kane’s Framework. Faculty-raters and standardized patients (SPs) used their respective InCoPrA sub-component to assess distinctive domains pertinent to ICS and Professionalism through multiple expert-built simulated-scenarios comparable to usual care. Evaluations included; inter-rater reliability of the faculty total score; the correlation between the total score by the SPs; and the average of the total score by two-faculty members. Participants were surveyed regarding acceptability, realism, and applicability of this experience.

**Results:**

Eighty trainees and 25 faculty-raters from five medical residency training sites participated. ICC of the total score between faculty-raters was generally moderate (ICC range 0.44–0.58). There was on average a moderate linear relationship between the SPs and faculty total scores (Pearson correlations range 0.23–0.44). Majority of participants ascertained receiving a meaningful, immediate, and comprehensive patient-faculty feedback.

**Conclusions:**

This work substantiated that InCoPrA was a reliable, standardized, evidence-based, and user-friendly assessment tool for ICS and Professionalism milestones. Validating InCoPrA showed generally-moderate agreeability and high acceptability. Using InCoPrA also promoted engaging all stakeholders in medical education and training–faculty, learners, and SPs—using simulation-media as pathway for comprehensive feedback of milestones growth.

## Background

Competency-based medical education (CBME) has become the cornerstone model of education and training in the U.S. and beyond [1, 2]. The Accreditation Council for Graduate Medical Education (ACGME) implemented the six global core competencies system in 1999 [3] and later on revised these efforts by implementing additional milestones in 2013 [4]. During those phases, educators have frequently struggled to find ways to evaluate learners’ skills in these fundamental areas. Although the ACGME produced the milestones to provide a framework for assessment [5–8], they tend to be subjective with language that allows room for interpretation, which likely reduces the fidelity and reliability of the milestones from one program or even one assessor to the other [9–11]. These competencies and milestones have also created an additional burden to already-overwhelmed educators and core faculty who genuinely want to spend sufficient time to properly teach and assess their trainee’s achievement of the competencies and professional growth [9, 12–14].

Since milestone reporting is required and is indirectly used to assess the quality of individual training programs, residencies are always searching for reliable, user-friendly, and efficient simplified assessment tools. Of the six competencies and their milestones, Interpersonal and Communication Skills (ICS) and Professionalism [15] have been particularly challenging to evaluate since they can be influenced by numerous factors [16].

The assessment of ICS and Professionalism has been studied using various methods of direct observation, global assessment, or Objective Structured Clinical Examinations (OSCEs), singularly or combined [17–23]. Methods that include simulation training and the use of Standardized Patients (SPs) are particularly important within this context [24–27]. In further attempts to improve the reliability of evaluations, others have used composite scores, checklist forms, and global rating scales within direct observation or simulation settings [20, 23, 28–39]. Despite these attempts, recent evidence suggests that the current available tools for medical education evaluation lack or provide insufficient validity evidence about their direction, value, educational outcome; thus limiting providing an intrinsic meaning and support decision making [40], and allowing room for improvements [13, 26, 41–43].

Validity is a growing science that has been widely studied using different approaches to provide a meaningful interpretation of an “output” to guide in decision making [26, 37, 42, 44–46].. The concept of validity has evolved over the last two decades [47–49]; most of the studies that assessed competencies and milestones did not sufficiently outline or adhere to validity frameworks to ascertain their findings [26, 37, 42, 44–46, 50]. Abu Dabrh et al., previously co-developed and piloted the *Instrument for Communication skills and Professionalism Assessment* (InCoPrA), a de novo tool used during an OSCE-like simulated training scenario to assess ICS and Professionalism [24]. The instrument showed strong feasibility and applicability within a residency training program setting; thus providing rational to further validate its use within other programs with larger participation using a contemporary approach to its validity evidence [33, 40, 51].

We hypothesize that the InCoPrA is a feasible, acceptable and reliable method to provide a meaningful and supportive validated interpretation of ICS and Professionalism skills of the learners, and to minimize the administrative burden of assessing their milestones using simulation settings. Generating such knowledge it will help minimizing the gap in evidence about validated assessment tools of these challenging competencies and milestones.

## Methods

The conceptualization and feasibility assessment of InCoPrA has been previously studied [24].

### Setting

The study occurred across five Department of Family Medicine (DoFM) sites at Mayo Clinic (Florida, Minnesota, Arizona, and Wisconsin) at their designated SimCenters. The study included medical learners from these participating sites who participated in the simulation activities. These activities were part of the expected didactic training and curricula; therefore, there were no special sampling methods of participants. The learners were not informed about the purpose of this scenario in order to minimize reactive biases. Each scenario was directly supervised by faculty (raters/assessors) in the respective SimCenters. Each simulation was video-recorded and all EMIR visited by participants during the encounter were live-tracked, recorded, and stored using a secure server with a password-protected data repository. The SimCenters staff created matching electronic medical records (EMR) and access to electronic medical information resources (EMIR) environment; thus, allowing participants to resources comparable to those in their routine practice.

### Participants

#### Observing faculty and SPs

All participating core faculty (assessors/raters) and SPs received standardized orientation about the proposed simulation activities and scenarios, debriefing techniques, and the use of performance checklist and global assessment on the InCoPrA. All faculty independently observed a scenario in real time, was blinded to other raters, and provided an evaluation at the conclusion of OSCE to the participants using the InCoPrA. The participating SPs had extensive experience in role-playing multiple patient scenarios for the SimCenters.

#### Medical learners

The study recruited mainly first-post-graduate year (PGY1), second- (PGY2) and third-year (PGY3) Family Medicine residents, and 3rd and 4th year medical students doing their clerkships at the DoFM. Learners were blinded to the scenarios they were administered during their simulation day experience.

### Educational intervention

The InCoPrA was developed, reviewed, and pilot-tested previously, taking into consideration the ACGME definition of competencies and existing tools used for other OSCE scenarios and competencies evaluation [52–54] and the feedback provided during the pilot testing [24]. The InCoPrA constructs assess professionalism if the trainee; 1) demonstrates integrity and ethical behavior; 2) accepts responsibility and follows through on tasks; and 3) demonstrates empathic care for patients. ICS was assessed through; 1) ability to utilize EMR and EMIR to understand the scenario; 2) communicate findings effectively with patients; and 3) communicate effectively with other healthcare professionals. The InCoPrA has three parts; The first two components, the *faculty and SP parts****,*** both use a 3-point Likert-like scale (outstanding; satisfactory; and unsatisfactory) and checklist with different questions (points) to address six categories/domains (the context of discussion, communication and detection the assigned task, management of the task assigned, empathy, use of EMR and EMIR, and a global rating); The 3rd component, ***the*** *participants’ self-evaluation survey* (REDcap® format), consisted of asking to: self-rate their performance after the encounter; self-rate their general skills of EMR and EMIR use; how realistic and acceptable the simulations felt; and how often they receive faculty feedback during their current training.

### Simulation scenarios

Building on our previous work [24], we have developed four scenarios which have been reviewed for content, realism, acceptability and expert validity by participating faculty members, leadership, SPs and non-participating learners. These scenarios were pertinent to: 1) detection of medical error [24, 55, 56]; 2) managing chronic opioid use; 3) Managing depression; and 4) Delivering bad news. In all these scenarios, the learners had access to a simulated EMR and EMIRs to help in identifying medical history, medication use, interactions and side effects. Before seeing the patient, the learners were instructed to perform an initial history, relevant exam (if needed), and discuss their findings with the patient. They also knew that they can leave the room to discuss the patient with faculty and then return to the room to dismiss the patient and discuss the plan of action. Afterwards, they debriefed with faculty.

### Validity approach

Our study design was guided by the Kane’s Framework as proposed by M. T. Kane [44, 57] and highlighted by others [47]; this approach proposes that to support validation assumption, studies should identify four critical inferences: 1) Scoring (i.e., to render the observed experiment into a quantitative or qualitative scoring system; 2) Generalization (i.e., to translate these scoring systems or scales developed into a meaningful general/overall interpretation of the performance; 3) Extrapolation (i.e., imply how/what this generalized inference translates into the real-world setting /experience; and 4) Implication (i.e., draw a final conclusion and reach a decision regarding its value/results).

In each respective site, the faculty-raters directly observed while using the InCoPrA to check-point and use the narrative interaction between learners and SPs and evaluated learners through their simulation encounter with SPs. (i.e. scoring). Each trainee went through 3 scenarios of various difficulties to allow sufficient spectrum for reproducibility and to minimize the error margin due to variation in performance. Each trainee was assessed by two raters. At the end, the faculty formulated their feedback through graded scale and narratives (i.e. generalization). Raters then had the opportunity to review and outline the performance of learners and compare that with the respective trainee’s real-world daily performance and pertinent scores (e.g. current milestones assessment reviews, course, internship … etc.) and draw conclusions and feedback to be provided to learners in person (i.e. extrapolation). Once these conclusions are reached, raters had a “sense of direction and action” to how the trainee performed and provided recommendations. For example, those learners who were observed and concluded to not have achieved well (i.e. unsatisfactory), faculty provided additional narrative feedback to outline the areas of needed improvement and identify deficiencies (i.e. implication).

Once these inferences from the Kane’s framework were synthesized, we used construct validity to evaluate evidence of validity [58]; construct validity demonstrates whether one can establish inferences about test results related to the constructs being studied. To test that, we compared all inferences with the current standing and evaluations of participants (i.e. convergence validity testing). Convergence construct validity testing compares the evaluated tool or instrument to others that are established. In this study, we compared InCoPrA results to the ACGME-proposed evaluation forms used routinely in the respective residency programs).

### Ethics approval and consent to participate

The study activities were approved by the Institutional Review Board (IRB) at Mayo Clinic as an educational intervention required and expected by the regular, didactic training and activities within the respective participating residency programs, and thus considered it to be IRB-exempt study (45 CFR 46.101, item 1); therefore no specific participation consent deemed required. The Mayo Clinic Simulation Center obtained standard consents for observation for all trainees per regular institutional guidelines. Additionally, the study authors and coordinators obtained standard consent for observation for all trainees per institutional guidelines.

### Statistical analysis

Each site was be randomized to 3 of the 4 scenarios by the study statistician; the order of the scenarios was also randomly assigned for each trainee. All analyses were done separately for each scenario. We descriptively summarized the percentage of learners with a satisfactory or outstanding rating separately according to rater and domain. We assigned points for each of the 6 domains with (unsatisfactory rating = 0; satisfactory rating = 1 point; and outstanding rating = 2 points). For each trainee, we calculated a total score by the standardized patient and a total score by each of the two faculty evaluators (scoring). Total scores were calculated by summing the responses of the 5 domains with a plausible score ranging from 5 to 15 (a lower score indicates a better performance). If a rating was missing for one of the domains, the mean of the other 4 domains was imputed for purposes of calculating the total score.

We descriptively summarized the standardized patients’ responses to each domain separately for each scenario. We evaluated the interrater reliability of the faculty total score with the intraclass correlation coefficient (Type 1) as described by Shrout and Fleiss [59] where faculty raters were assumed to be randomly assigned (generalization). For each domain, we assessed interrater reliability using the weighted kappa statistic. We additionally evaluated the correlation between the total score by the standardized patient and the average of the total score by the two faculty members (extrapolation). Kendall rank correlation coefficients will examine the correlation between SP assessment total raw score, the consensus faculty assessment total raw score, and the ACGME milestone evaluations for ICS and professionalism, separately for each scenario (implication).

## Results

Eighty learners were included from five different sites. The scenarios assigned to each center are shown in Appendix Table 5. Table 1 summarizes the rationale, methods, and findings as guided by the Kane’s Framework from this study.

**Table 1:** Summary of rationale, methods, and findings as guided by the Kane’s Framework for validity during study course using InCoPrA

Steps	Rationale	Methods	Results
Scoring	Render the observed experiment into a quantitative or qualitative scoring system	Direct observations and assessments by faculty and SPs using InCoPrA	Table 2
Generalization	Translate these scoring systems or scales developed into a meaningful general/overall interpretation of the performance	Meaningful feedback formulation through InCoPrA’s graded and nominal scale and narratives	Tables 2 & 3
Extrapolation	Imply how/what this generalized inference translates into the real-world setting /experience	Comparing and discussing simulation feedback to real-life performance feedback	Table 4
Implication	Draw a final conclusion and reach a decision regarding its value/results	Faculty providing next-steps direction and recommendations to support milestones growth and progress	Table 4-guided Verbal discussions ACGME-supported Evaluation forms and mentored discussions

*InCoPrA* Instrument for Communication skills and Professionalism Assessment; *SP* Standardized Patients; *ACGME* Accreditation Council for Graduate Medical Education

The trainee assessment by the standardized patient is summarized in Table 2. The median (interquartile range) for the total scores indicated satisfactory to outstanding performance by most learners [Scenario A: 6 (5–8); Scenario B: 5 (5–7); Scenario C: 7 (6–8); Scenario D: 5 (5–7)].

**Table 2:** Assessment by Standardized Patient according to Simulation Scenario

	Simulation Scenario			
Domain	A (***N*** = 57)	B (***N*** = 64)	C (***N*** = 45)	D (***N*** = 65)
**Ability to explain the facts regarding the proposed task?**				
1 = Outstanding	33 (60%)	50 (78%)	19 (42%)	42 (70%)
2 = Satisfactory	20 (36%)	14 (22%)	25 (56%)	18 (30%)
3 = Unsatisfactory	2 (4%)	0 (0%)	1 (2%)	0 (0%)
Not reported	*N* = 2	*N* = 0	*N* = 0	*N* = 5
**Honesty and truthfulness**				
1 = Outstanding	47 (82%)	56 (88%)	32 (71%)	56 (90%)
2 = Satisfactory	10 (18%)	8 (13%)	13 (29%)	6 (10%)
3 = Unsatisfactory	0 (0%)	0 (0%)	0 (0%)	0 (0%)
Not reported	*N* = 0	*N* = 0	*N* = 0	*N* = 3
**Empathy**				
1 = Outstanding	36 (64%)	43 (67%)	28 (62%)	38 (61%)
2 = Satisfactory	20 (36%)	21 (33%)	16 (36%)	24 (39%)
3 = Unsatisfactory	0 (0%)	0 (0%)	1 (2%)	0 (0%)
Not reported	*N* = 1	*N* = 0	*N* = 0	*N* = 3
**Providing closure to the discussion**				
1 = Outstanding	39 (70%)	53 (84%)	21 (49%)	48 (76%)
2 = Satisfactory	17 (30%)	10 (16%)	22 (51%)	15 (24%)
3 = Unsatisfactory	0 (0%)	0 (0%)	0 (0%)	0 (0%)
Not reported	*N* = 1	*N* = 1	*N* = 2	*N* = 2
**Comfort with entrusting a loved one’s care to this learner**				
1 = Comfortable	43 (78%)	59 (92%)	39 (87%)	58 (91%)
2 = Somewhat comfortable	12 (22%)	5 (8%)	5 (11%)	6 (9%)
3 = Not at all comfortable	0 (0%)	0 (0%)	1 (2%)	0 (0%)
Not reported	*N* = 2	*N* = 0	*N* = 0	*N* = 1
**Total score**	6 (5, 5, 8, 11)	5 (5, 5, 7, 10)	7 (5, 6, 8, 12)	5 (5, 5, 7, 10)

Data are given as the number and percentage of trainees for individual domains and median (minimum, 25th percentile, 75th percentile, maximum) for the total score

Interrater reliability of the total score between faculty raters was generally moderate for the four simulation scenarios (ICC range 0.44 to 0.58, Table 2). Kappa scores for the individual domains are reported in Table 3.

**Table 3:** Interrater Reliability of Faculty Assessments

	Simulation Scenario			
Domain	A	B	C	D
**Communication**	0.47	0.23	0.26	0.40
**Context**	0.48	0.55	0.56	0.55
**Empathy**	0.45	0.23	0.27	0.26
**Management**	0.38	0.25	0.39	0.41
**Global**	0.50	0.41	0.65	0.53
**Total score**	0.58	0.44	0.53	0.50

The intra-class correlation coefficient is reported for the total score while the weighted kappa statistic is reported for the individual domains

**Fig. 1:**
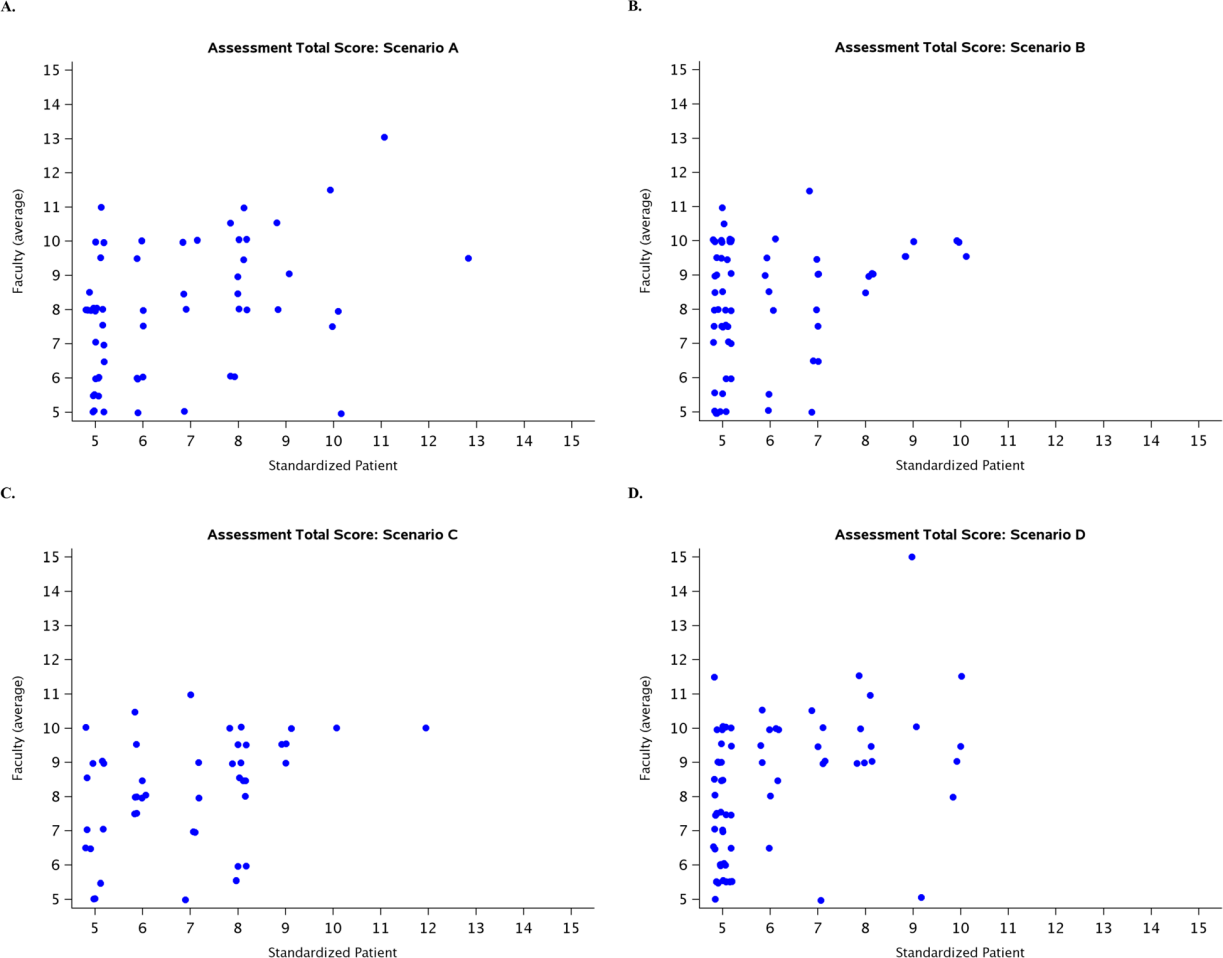
**a**: Relationship of Trainee Assessment between Standardized Patient and Faculty Reviewers (Scenario A). **b**: Relationship of Trainee Assessment between Standardized Patient and Faculty Reviewers (Scenario B). **c**: Relationship of Trainee Assessment between Standardized Patient and Faculty Reviewers (Scenario C). **d**: Relationship of Trainee Assessment between Standardized Patient and Faculty Reviewers (Scenario D)

There is a generally moderate linear relationship between the standardized patient and faculty total scores (Pearson correlations range from 0.23 to 0.44) (Table 3 and Fig. 1-a, b, c, and d). Rank based correlations (Kendall and Spearman) are additionally reported in Table 4.

**Table 4:** Correlation of Total Scores between Faculty Raters and Standardized Patient

Correlation	Simulation Scenario							
	A	***p*** value	B	***p*** value	C	***p*** value	D	***p*** value
**Kendall Tau**	0.28	0.007	0.16	0.11	0.34	0.003	0.39	< 0.001
**Spearman rank**	0.36	0.007	0.18	0.15	0.43	0.003	0.49	< 0.001
**Pearson**	0.41	0.002	0.23	0.065	0.40	0.006	0.44	< 0.001

Among the 78 learners, 71 completed a post-training survey. Their responses are summarized in Appendix Table 6.

## Discussion

Validating InCoPrA showed generally-moderate agreeability, high acceptability, and strong evidence of benefit and feasibility. Users found its standardized structure to be efficient, simplified, and user-friendly for assessment of ICS and Professionalism milestones.

Honest assessment of competency-based and milestones outcomes for learners is essential for professional growth and development [43, 45]. While many tools and checklists that assess clinical skills have been studied and described, very few have been validated [16, 37, 38, 42, 51]. In particular, assessment of professionalism and ICS is very complex and is highly influenced by raters [43, 49–51]. Teaching raters to use a tool is as simple idea, but is often overlooked in preparation for assessments [37, 43, 48, 57, 58]. Utilizing a validated, easy to use, easy to train tool like InCoPrA, can set the stage for fair assessment of professionalism and ICS and more importantly promote dialogue within the Clinical Competency Committee (CCC). This validation study was completed using 80 learners, 25 faculty-raters from five medical residency training sites, 12 SPs and 4 OSCE’s to validate InCoPrA as a feasible and user-friendly tool to be used when assessing professionalism and ICS. While 82% of learners who completed the post-training self-assessment scored the scenario as realistic, this has the potential to confound rater scores if the scenario is viewed as less realistic especially given the ICS and professionalism domains. 69% of learners who completed the post training self-assessment felt that more simulation training done in similar fashion could be beneficial.

Utilizing InCoPrA in an OSCE scenario is valuable because faculty-raters and SPs are able to view multiple learners in the same scenario, allowing for richer feedback. Including SPs as part of the assessment team gives an additional nuanced and contextual perspective of all stakeholders. While the data shows generally moderate agreeability between faculty-raters and SPs, there were observed differences in other ratings and scoring, with more positive skewness from the SPs. While this could be due to with rater nuances, however, skewness or variations in assessment and perception between physicians and patients– as observed by the SPs ratings here— is not a novel phenomenon, and it agrees with previous findings [60–65]. This phenomenon could be explained by the innate variation in ‘performance perception and assessment’ between patients and educators/physicians due to the nature and significance of their specific roles. Patients often emphasize on and identify compassionate and positive interactions as surrogate of quality care and value while educators/physicians especially focus more on the clinical knowledge and management skills [60, 61, 66]. The varied interdependence and difference in scoring can serve as a prompt for the CCC to create a space for an open dialogue regarding these potential differences. Future studies may also need to better define the scoring differences and its important role, in line with other reports [64, 67, 68].

### Strengths and Limitations

To overcome barriers often encountered in validity studying, we used evidence-based validity framework to; guide our study design, filling the gap in current evidence [47, 51]; employ different scenarios with various levels of difficulties; use currently-adopted forms of evaluation to compare findings; include expert/core faculty raters; and included portals to deliver and receive feedback between learners and faculty. Performance contamination may occurred if learners took the OSCE and then informed other learners of upcoming scenarios, though previous research found that using such study methodology did not result in significant differences in performance among their tested learners [69]; however, we instructed all learners to avoid sharing their experiences. Most of the learners and faculty represent the specialty of Family Medicine. Further studying of InCoPrA will be needed to define the generalizability as it pertains to discipline and setting (simulation versus clinical). We also realize that generalization in our study to other institutions might be limited by availability of resources, faculty training and IT infrastructure provided at Mayo Clinic; however, these activities may still be conducted within resource-limited that incorporates faculty-SP-learner by modifying the simulation center resources to direct OSCE-style setting.

## Conclusions

Existing comparable assessment tools lack sufficient validity evidence, direction, and educational outcomes. This work examined these gaps by substantiating that InCoPrA is a standardized, evidence-based, user-friendly, feasible, and competency/milestone-specific assessment tool for ICS and Professionalism. Validating InCoPrA showed a generally moderate agreeability, and high acceptability and strong evidence of benefit. Using InCoPrA also promoted engaging all stakeholders in medical education and training –faculty, learners, and SPs—through using simulation-media as pathway for comprehensive feedback of milestones growth. In addition to the importance of education and training provided by faculty, engaging patients in providing feedback about Professionalism and ICS of medical learners is valuable to assess and improve these core competencies and milestones. Allowing immediate reflective feedback from learners also enhances this comprehensive-feedback approach as shown through using InCoPrA.

## Data Availability

The datasets used and/or analyzed during the current study are available from the corresponding author on reasonable request.

## Appendix

**Table 5:** Scenarios Assigned to Each Site

	Simulation Scenario^**a**^			
Site	A	B	C	D
**Kasson/Rochester, MN**	X	X		X
**Jacksonville, FL**	X		X	X
**La Crosse, WI**		X	X	X
**Eau Claire, WI**	X	X	X	

^**a**^A = Detection of medical error, B = Managing chronic opioid use, C = Managing depression, D = Delivering bad news

**Table 6:** Trainee Self-Assessment Responses

	Summary (*N* = 71 trainee respondents)
**Campus Location:**	
Mayo Clinic Florida	12 (17%)
Mayo Clinic Rochester	28 (39%)
Mayo Clinic La Crosse, WI	21 (30%)
Mayo Clinic Eau Claire, WI	10 (14%)
**1. How would you generally rate your own generalskills in your clinical work?**	
Unsatisfactory	1 (1%)
Satisfactory	67 (94%)
Outstanding	3 (4%)
**2. How would you rate your own performance on using available electronic resources in your daily practice?**	
Unsatisfactory	4 (6%)
Satisfactory	60 (85%)
Outstanding	6 (8%)
Not reported	1 (1%)
**3. How realistic were the scenarios today?**	
Somewhat realistic; it could have been better	13 (18%)
Realistic; I did or may experience similar encounter this in my practice	58 (82%)
**4. How would you rate your comfort level with using the computer to access various electronic resources in your daily practice?**	
Unsatisfactory	5 (7%)
Satisfactory	54 (76%)
Outstanding	12 (17%)
**5. How often have you received feedback from someone, such as a Staff member or more senior resident, on your use of electronic medical resources?**	
Never	18 (25%)
Sometimes	47 (66%)
Always	6 (8%)
**6. Have you received sufficient training in using electronic medical resources?**	
No	6 (8%)
Yes	65 (92%)
**7. If yes, at what level did this occur?**	N = 65
Medical School	3 (5%)
Residency	17 (26%)
Both	42 (65%)
Not reported	3 (5%)
**8. Do you think it would be helpful to receive more electronic medical resources training?**	
Missing	2
No	25 (36%)
Yes	44 (64%)
**9. If yes, at what level would this be most helpful?**	
Missing	27
Medical School	2 (5%)
Residency	19 (43%)
Both	23 (52%)
**10. Would you like to see more simulated training like this in your program?**	
Missing	6
No	20 (31%)
Yes	45 (69%)

## Abbreviations

InCoPrA: Instrument for Communication skills and Professionalism Assessment
DoFM: Department of Family Medicine
CCC: Clinical Competency Committee
ACGME: Accreditation Council for Graduate Medical Education
CBME: Competency-based medical education
ICS: Interpersonal and Communication Skills
SPs: Standardized Patients
EMR: Electronic Medical Records
EMIR: Electronic Medical Information Resources
PGY: Post-Graduate Year
OSCE: Objective Structured Clinical Examination
